# Exosomes of Whartons’ jelly mesenchymal stem cell reduce the NOX genes in TGF-β-induced hepatic fibrosis

**DOI:** 10.22038/IJBMS.2022.66802.14649

**Published:** 2022-12

**Authors:** Reza Afarin, Tahereh Behdarvand, Elham Shakerian, Samaneh Salehipour Bavarsad, Mojtaba Rashidi

**Affiliations:** 1 Cellular and Molecular Research Center, Medical Basic Sciences Research Institute, Ahvaz Jundishapur University of Medical Sciences, Ahvaz, Iran; 2 Department of Clinical Biochemistry, Faculty of Medicine, Jundishapour University of Medical Sciences, Ahvaz, Iran

**Keywords:** Exosome, HSCs/LX2, Liver fibrosis, Mesenchymal stem cells, NADPH oxidase, TGF β/Smad3C, Whartons’ jelly

## Abstract

**Objective(s):**

Activated cells which are called star-shaped cells, are some of the key factors in the development of liver fibrosis. Activation of NADPH oxidase (NOX) is associated with increased HSCs activity and progression of hepatic fibrosis. In this study, the effects of human exosomes derived from WJ-MSCs on NOX1, NOX2, and NOX4 gene expression in TGF-β-induced hepatic fibrosis were investigated.

**Materials and Methods:**

LX2 cell line was treated with 2 ng/ml TGF-β for 24 hr, in order to induce liver fibrosis after starvation. In the next step, the cells were treated with several concentrations of the exosomes derived from WJ-MSCs (10, 20, 30, 40, and 50 μg/ml). Finally, Smad3C phosphorylated protein expression level and NOX1, NOX2, and NOX4 gene expression levels were measured.

**Results:**

The results demonstrated that the level of NOX1, NOX2, and NOX4 mRNA expressions decreased significantly during 24 hrs at concentrations of 40 and 50 μg/ml of WJ-MSCs exosomes in TGF-β-induced-HSCs. The p-Smad3C proteins were significantly decreased (fold change: 1.83, *P-value<*0.05) after exposure to WJ-MSC-derived exosomes.

**Conclusion:**

Treatment with exosomes prevents further activation of HSCs by inhibiting the level of Smad3C phosphorylation. The experimental data of our study suggested that in liver fibrosis, the protection of HSCs activation against TGF-β by inhibiting the NOX pathway via human exosomes of WJ-MSCs is extremely important. It needs further research as a treatment method.

## Introduction

Most chronic liver damages such as excessive alcohol consumption, nonalcoholic fatty liver disease (NASH) (1, 2), autoimmune diseases, and drug effects activate hepatic stellate cells (HSCs) and increase the production and accumulation of extracellular matrix (ECM) proteins, which ultimately lead to liver fibrosis (1). The progression of liver fibrosis leads to cirrhosis and liver failure. The most important clinical consequences of cirrhosis are liver dysfunction and hepatocellular carcinoma (HCC). HSCs are activated by inflammatory cytokines, chemokines, growth factors, and oxidative stress products (ROSs) (3, 4). Therefore, new therapeutic strategies are needed to inhibit the activated HSCs in order to treat and prevent the progression of liver fibrosis (5, 6).

Nicotinamide adenine dinucleotide phosphate (NADPH) oxidases (NOXs) play an important role in the development of a variety of diseases, including liver disease, by producing ROSs. HSCs are some of the main NOX-producing cells. HSCs produce NOX1, NOX2, and NOX4, which play substantial roles in the process and development of liver fibrosis (7). TGF-β (7), Angiotensin II (8), and PDGF produce NOXs to enhance HSC proliferation and exacerbation of liver fibrosis. Among them, TGF-β plays an important role in inducing the TGF-β/Smad signaling pathway, especially Smad3 (9, 10). Smad3 acts as a signaling mediator for TGF-β (11). TGF-β binds to its serine /threonine kinase receptors that activate the TGF-β signaling pathway. TGF-β receptor type II phosphorylation by TGF-β1 leads to phosphorylation and activation of TGF-β receptors type I, which in turn phosphorylates R-Smad proteins (Smad2/3). R-Smad then forms a heterocomplex with Smad4 (Co-Smad), which is eventually translocated into the nucleus to regulate gene transcription (12), such as NOXs and other extracellular matrix proteins (13). Therefore, one way to prevent HSC activation and reduce hepatic fibrosis is to inhibit activated HSCs by stopping the TGF-β/Smad3 signaling pathway, which is considered a potential therapeutic target (14-16). 

Recently, the use of stem cell therapy has been suggested as an effective alternative treatment for liver fibrosis (17), through tissue repair and immune system regulation (18). 

Recently, the therapeutic effects of mesenchymal stem cells (MSCs) are attributed to paracrine actions (18). Exosomes are extracellular microvesicles that are mainly characterized by a diameter of 30 to 100 nm. These vesicles contain proteins, nucleic acids (e.g., DNA, mRNAs, and miRNAs), and lipids (19). Wharton’s jelly-derived MSCs (WJ-MSCs) are interesting candidates for cell therapy (20).

The effects of MSCs-derived exosomes are mediated by modulating the immune system and inhibiting the release of many inflammatory factors (21). There are several advantages to using exosomes as a cell-free therapy that can replace conventional cell therapy (22), such as lower immunogenicity, prevention of complications due to MSCs-induced tumor formation, and immune rejection (23). The main purpose of this research was to investigate the effects of exosomes of WJ-MSC on NOX gene expression levels and phosphorylation of Smad3 protein in TGF-β-induced liver fibrosis.

## Materials and Methods

***Reagents***

TGF-β and RIPA buffer were purchased from Sigma-Aldrich (St. Louis, MO, USA). PVDF membranes were purchased from Millipore (USA). Fetal bovine serum (FBS) was purchased from Gibco. Trypsin-EDTA and pen/strep antibiotics were purchased from Ideazist (IRAN). PE-conjugated mouse anti-human (CD34, CD45, CD44, and CD105) antibodies were purchased from eBioscience. EXOCIB isolation kit purchased from CIB Biotech Co. (Iran).

***Isolation and culture of WJ-MSCs***

Prior to the delivery of the infants, informed consent was obtained from the mothers. The fresh human umbilical cord was removed from the mother after full-term cesarean delivery. By washing the umbilical cord isolated from the neonates with phosphate buffer solution (PBS), its blood vessels were removed. Wharton jelly was then cut into pieces of 2-3 mm^3^ and placed in a flask with a complete culture medium composed of low glucose DMEM with 20% FBS and 100 U/L penicillin-streptomycin. The explant pieces were incubated at 37 ^°^C with 5% CO_2_. After three to five days, non-adherent cells were removed from the supernatant of MSCs. The supernatant of the MSCs was changed once every two days until the cells reached a confluency of 80%. After 2 weeks, the WJ-MSCs reached confluency for the first passage. The cell culture medium was changed every 3 days.

***Di*** ***ﬀ*** ***erentiation assays of WJ-MSCs***

WJ-MSCs from passage 3 were used for all experiments. For MSC differentiation, 50,000 cells/ml were implanted in a 6-well plate. Then, the cells were exposed to osteogenic and adipogenic growth factors in a medium containing 10% FBS for 3 weeks. After fixing the cells with 10% formalin, the cells were stained with Alizarin red solution and Oil Red O to differentiate osteogenic and adipogenic differentiation, respectively.

***Detection of WJ-MSCs surface markers***

After the second passage, WJ-MSCs were trypsinized. Following washing the cells with PBS, they were stained with monoclonal antibodies according to the manufacturer’s recommendation: PE- CD34, PE- CD45, PE- CD44, and PE- CD105. The required extracted cells were examined using the well-known flow cytometry technique (Becton Dickinson, USA), and finally, the data was evaluated using FlowJo analyzer software.

***Exosomes isolation***

To isolate exosomes derived from WJ-MSCs, the FBS concentration was reduced gradually; this means that approximately every 3 days, the supernatant medium of WJ-MSCs was replaced with a medium containing less FBS (the FBS of the medium was reduced by 2% each time). Finally, the cells were exposed to the FBS-free medium. After that, the cell culture-conditioned media was collected after 3 days, and the supernatant was collected and passed through a 0.22 μm filter and centrifuged at 3000 rpm for 10 min. The exosomes were then isolated according to the manufacturer’s instructions for the EXOCIB isolation kit (CIB Biotech Co., Iran) and placed at -80 ^°^C. Proteins isolated from the exosome were examined for their concentration using the BCA method (Parstous, Iran). Primary antibodies against P-Smad3 (Ser423/425, 1:2000) and GAPDH 1:500; (Abcam, UK) were used for western blot. 

***Cultivation and treatment of HSCs***

LX-2, an immortalized human HSC cell line (a gift from Professor S. Friedman) was cultured in a 6-cell plate with 1×10^5^ cells/well in DMEM and placed at 37 ^°^C with 5% CO_2_ (24). Then, the cells were treated for 24 hr, with 2 ng/ml TGF-β (25). Next, the concentrations of exosomes, including 10, 20, 30, 40, and 50 μg/ml were dissolved in a culture media (free-FBS) and added to the cells for 24 hr. Three groups were considered for the experiment: 1-control group (without treatment), 2-TGF-β treatment group, and 3-TGF-β treatment group with different concentrations of exosomes.

***Characterization of exosome***

Exosomes were characterized by TEM and DLS. For TEM, after fixing the exosomes with glutaraldehyde, they washed them with PBS and ethanol which was used to dehydrate the samples. After evaporating the ethanol, the samples were kept at room temperature and allowed 24 hr to dry (26). For DLS, the exosome content solution was diluted to 1 μg/ml in PBS. Finally, the size of the extracted exosomes was evaluated by DLS Zetasizer Nano (Malvern Corp) at 23 ^°^C according to the manufacturer’s instructions (27).

***Real-time PCR technique***

After collecting the cells, the total RNA was extracted with a Qiagen extraction kit (Germany) according to the manufacturer’s instructions. Subsequently, cDNA synthesis was performed with PrimeScript™ RT cDNA synthesis kit (Takara, Japan), Rox SYBR Green Master (Amplicon, Denmark) was used to perform the real-time PCr technique in order to determine the expression of NOX1, NOX2, NOX4 genes. GAPDH was used to normalize gene expression as an internal control. Primers used for real-time PCR technique were: 

NOX1 (F: 5′-CTGTTGCCTAGAAGGGCTCC-3′,

R: 5′-ACAGGCCAATGTTGACCCAA-3′),

NOX2 (F: 5′-GTTGCCCGAGATGCCAATTC-3′,

R: 5′-CATGTCCAGGAATCGCTCCA-3′),

NOX4 (F: 5′-TGGAGGAAGAGGGAAGAGGT-3′,

R:5′-AGAGCCAGATGAACCCAAGC-3′),

GAPDH (F:5′-TTCACCACCATGGAGAAGGC--3′,

R:5′-GGCATGGACTGTGGTCATGA-3′).

***Detection of ROS Production***

ROS assay was performed using the fluorimetric method and the 2′, 7′-dichlorodihydrofluorescein (H2DCF) probe. The basis of this method is the conversion of H2DCF to 2%, 7′-dichlorofluorescein (DCF) by the amount of hydrogen peroxide (H2O2) produced in the cell. The DCF is measured by fluorescence light, and the reflected light was measured in the range of 500–600 nm. For this purpose, 5×10^4^ cells/well were cultured in 12-well cell plates for 24 hr, the HSCs were treated with TGF-β (2 ng/ml) for 24 hr, and then, the cells treated with different concentrations of WJ-MSCs exosomes (10, 20, 30, 40, and 50 μg/ml). After washing the cells with PBS at the end of the incubation time, 25 μM of H2DCF solution was added to each well and placed at 37 ^°^C for 30 min. At the end of 30 min, fluorescence light was measured at 488 nm excitation and the emission in the range of 500-600 nm.

***Western blotting technique***

To perform the western blotting technique, after digesting the HSCs cells with trypsin-EDTA, they were lysed with RIPA lysis buffer in an ice bath. Protein bands were then isolated on SDS-PAGE and after transfer to the PVDF membrane, blocking was performed, and finally, the membrane was adjacent to primary and secondary antibodies. Finally, using the ECL reagent, band detection was performed on a ChemiDoc device.

***Statistical analysis***

The results were analyzed as (Means±SEM) by ANOVA and Tukey tests using GraphPad Prism 9.0.1 software. A *P*-value less than 0.05 was considered significant.

## Results

***Characterization of WJ-MSCs***

**Figure 1:**
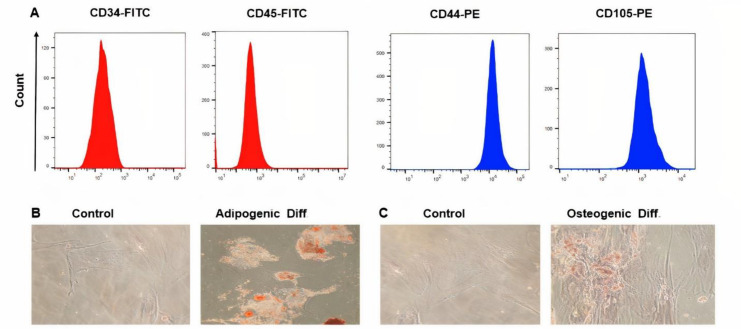
Immunophenotyping and differentiation potentials of WJ-MSCs. (A) WJ-MSCs in the Flow cytometry system, express CD44 and CD105, and are negative CD34 and CD45 markers. (B) Oil Red O staining of WJ-MSCs (C) Alizarin Red S staining of WJ-MSCs

We used human WJ-MSCs isolated from the umbilical cord after the third passage, which are presented as monolayer fibroblast-like and spindle-shaped adherent cells. The WJ-MSCs were positive for (CD44 and CD105), but were negative for (CD34 and CD45), which indicates that the isolated cells do not originate from endothelial and hematopoietic cells (Figure 1A). To confirm the potential of differentiation of WJ-MSCs *in vitro*, after 21 days of treatment with a differentiating medium, lipid vacuoles in WJ-MSCs were observed with O red oil staining (Figure 1B). Moreover, after differentiation into osteoblasts, calcium deposits were observed with Alizarin red staining (Figure 1C).

***Exosome characterization***

**Figure 2:**
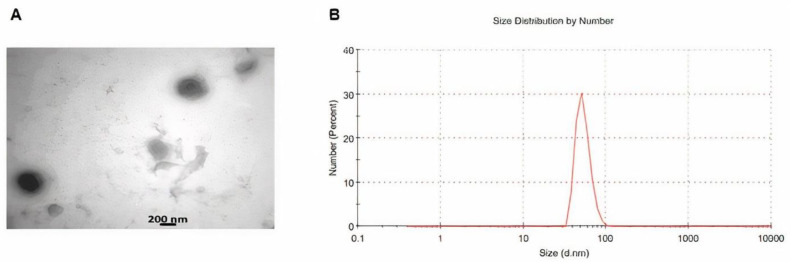
Characterization of exosomes. (A) TEM of the exosomes to visualize the shape and size of these vesicles. (B) Exosome size determination by Malvern zeta sizer. Up to 85% of total exosomes were 73 nm in diameter

TEM analysis exhibited that the exosomes had a spherical shape with a size between 50 and 200 nm (Figure 2A). The marker proteins of CD9 and CD81 were all expressed in exosomes with significantly higher levels than the WJ-MSC with western blot analysis (Figure 2B). The size of most isolated exosomes was 73 nm using a zeta sizer (Malvern Corp) (Figure 2C).

***NOXs mRNA expression in TGF-β-treated HSCs and exosomes***

**Figure 3:**
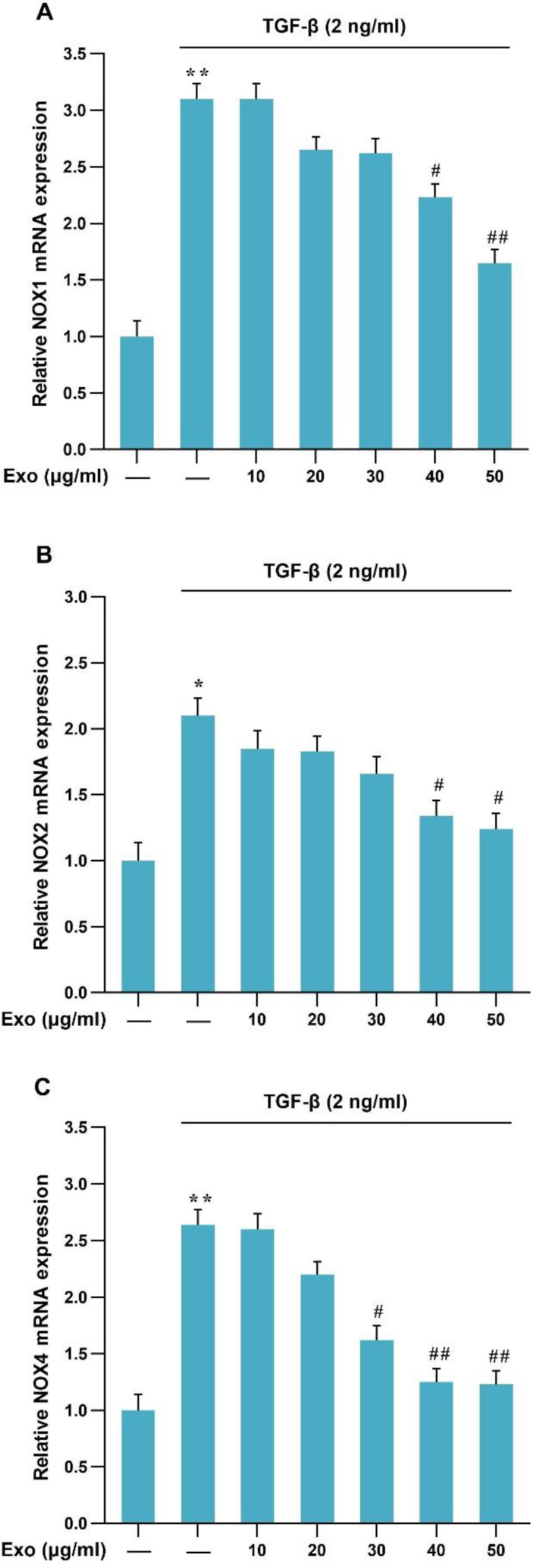
mRNA expression level of NOXs genes in the presence of TGF-β (2 ng/ml) and exosome in LX2 cells. Results of three replications (Mean±SEM) control have been reported. The significance level was considered *P<*0.05 (**P<0.01, ^#^*P<*0.05, ^##^*P<*0.01)

After treatment of LX-2 with TGF-β1 (2 ng/ml) for 24 hr, it was observed that the expression of NOX1, NOX2, and NOX4 mRNA increased compared with the control group. Then, TGF-β1-activated HSCs were treated at different concentrations of WJ-MSC exosomes (10, 20, 30, 40, and 50 μg/ml) for 24 hr. The results demonstrated that the level of NOX1, NOX2, and NOX4 mRNA expressions decreased significantly during 24 hr at concentrations of 40 and 50 μg/ml of WJ-MSCs exosomes in TGF-β-induced-HSCs compared with the other concentrations of exosomes (Figure 3A, B, and C).

***ROS production in TGF-β-treated HSCs and exosomes***

**Figure 4:**
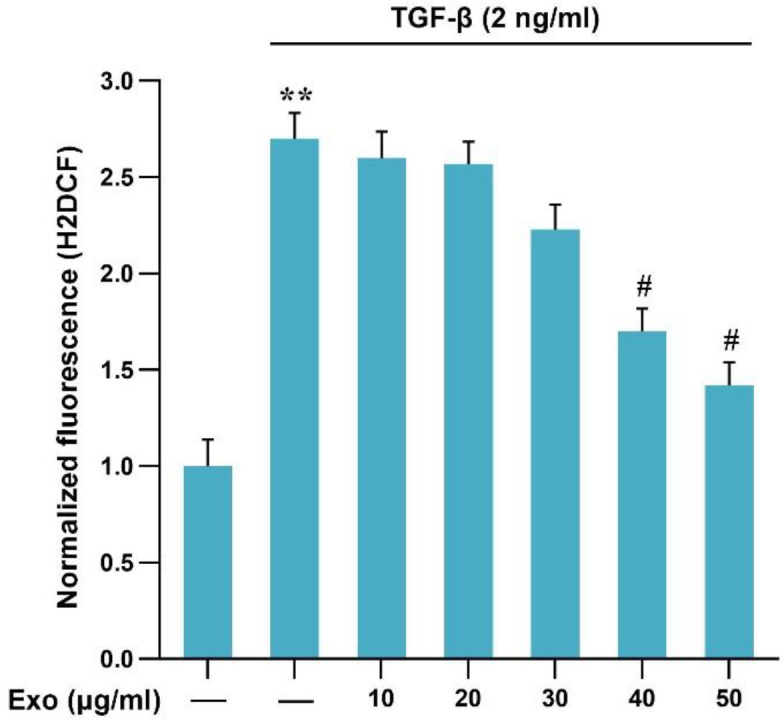
Detection of ROS production in the presence of TGF-β and exosomes in LX2 cell line: Results of three replications (Mean±SEM) control have been reported. The significance level was considered *P<*0.05 (**P<0.01, ^#^*P<*0.05)

To evaluate the production of ROS, after incubation of HSCs with TGF-β and the occurrence of liver fibrosis conditions, and finally treatment with different concentrations of exosomes of WJ-MSCs, ROS production was examined. ROS production was significantly increased by exposure to TGF-β but showed a significant decrease by treatment with concentrations of 40 and 50 μg/ml of exosomes of WJ-MSCs (Figure 4A).

***Phosphorylation of Smad3C protein in TGF-β-treated HSCs and exosomes***

**Figure 5:**
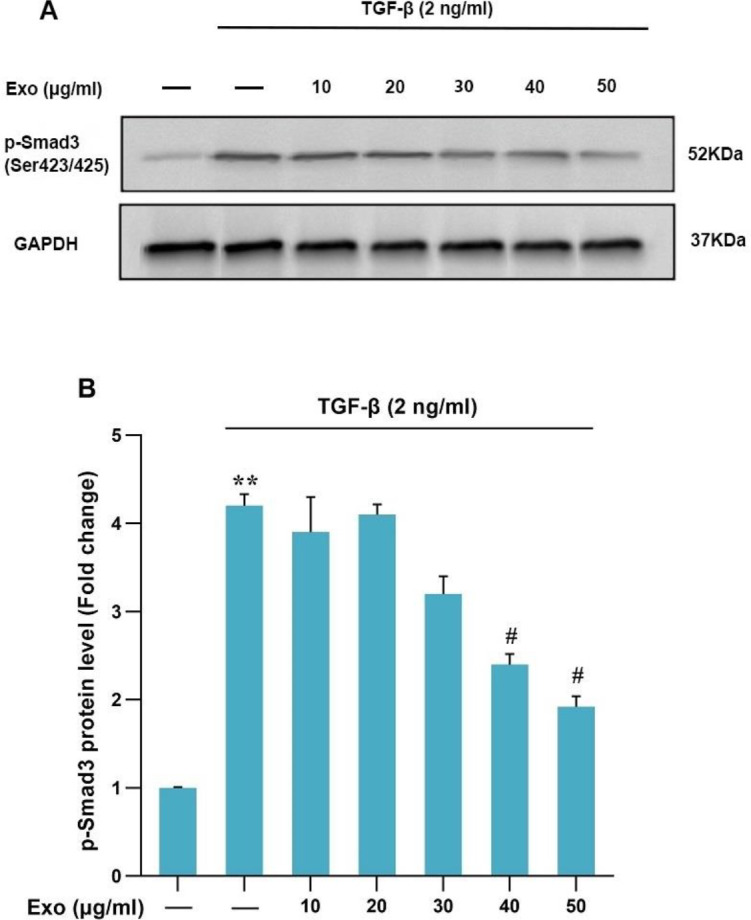
Evaluation of the effect of 4 hr exosome treatment on TGF-β-induced Smad3C phosphorylation in LX2 cell line: Results of three replications (Mean±SEM) were reported. The significance level was considered *P<*0.05. GAPDH protein was used as an internal control gene (**P<0.01, ^#^*P<*0.05)

To investigate the effect of exosomes of WJ-MSCs on the phosphorylation of Smad3C in TGF-β-induced HSCs, the first HSCs cells were treated with the exosomes of WJ-MSCs (10, 20, 30, 40, and 50 μg/ml) for 4 hr; then the cells (LX2-Cells) were treated with TGF-β (2 ng/ml) for 30 min. Western blot results confirmed that Smad3C phosphorylation in TGF-β-induced HSCs was significantly increased compared with the control group. In contrast, WJ-MSC-derived exosomes were able to significantly reduce Smad3C phosphorylation (Figures 5A and B).

## Discussion

Hepatic fibrosis occurs due to damage and death of liver cells and activation of HSCs. Any kind of damage to hepatocytes, such as hepatitis, excessive alcohol consumption, and iron overload, can cause ROS production from lipid peroxidation processes (28). In recent years, MSCs have received more attention in regenerative medicine because of their practical benefits (29). The WJ-MSCs have significant implications for regenerative medicine compared with other adult MSCs (30). Inhibition of activated HSCs is an approach to the improvement of liver fibrosis. In this regard, human WJ-MSCs are important clinical candidates in this field (31). On the other hand, MSCs may mediate a protective role by paracrine and autocrine signaling in liver fibrosis (32, 33). This research was performed to investigate the effect of exosomes of human WJ-MSCs on inhibition of the NOXs pathway in TGF-β-induced HSCs.

Evidence suggests that up-regulation of different types of NOXs is the main reason for the increase in intracellular ROS concentration in liver fibrogenesis (34). NOX mediates fibrogenic reactions in response to several agonists, including angiotensin II, PDGF, and TGF-β in HSCs. NOX-derived ROS is a major mediator in liver fibrogenesis as well as in other organs such as the kidneys, heart, and lungs (35). 

In this study, TGF-β-induced HSC cells increased gene expression of NOX subunits and ROS production compared with controls. A study by Lan *et al*. showed that reducing NOX1 and NOX4 leads to reduced liver fibrosis in carbon tetrachloride (CCl4)-treated mice (36). In animal models, the expression of NOX enzymes in HSCs leads to exacerbation of inflammation and liver fibrosis. The expression of NOX1 and NOX2 was increased in the mouse model of liver fibrosis with CCl4 and bile duct ligation (BDL). ROS production is reduced by eliminating NOX enzymes which lead to reduced inflammation and prevents liver fibrosis progression (37). Other studies have shown that the severity of liver fibrosis is associated with the expression of NOX enzymes in HSCs, which is related to the TGF-β/SMAD3 signaling pathway (10). 

However, in response to exosomes of WJ-MSCs treatment in TGF-β induced HSCs, the expression of NOX1, NOX2, and NOX4 enzymes and ROS production were down-regulated. Exosomes obtained from umbilical cord MSCs can restore the balance of oxidation and antioxidation both *in vivo* and *in vitro* (38). The use of exosomes of MSCs, unlike cell therapy, has no risks of cell therapy, such as aneuploidy and transplant rejection, and may provide an alternative therapy for liver fibrosis (22). On the other hand, the high levels of reactive oxygen/nitrogen species (RONS) at the injury site reduce MSC survival and therefore cause lack of secretion of the relevant cytokines and the loss of therapeutic effects (39). Bataller *et al*. showed that human BM-MSCs significantly reduced NOX activity in liver fibrosis (40). Previous studies have demonstrated that human WJ-MSC-derived exosomes can suppress NOX and ROS production in kidney tissue, which leads to inhibition of apoptosis and improvement of kidney function (41). In the model of ischemia-reperfusion (IR) injury in rats, the exosomes of adipose MSCs lead to the reduction of NOX1 and NOX2 levels, reduction of creatinine and BUN levels, and improvement of kidney function (42).

 However, given its effects on NOX activity, exosomes of WJ-MSCs treatment reduced phosphorylated Smad3C in TGF-β induced HSCs. Qiao *et al*. showed that the anti-fibrotic effect of human BMSCs to inhibit activated-HSCs and to induce their apoptosis is mediated through NADPH oxidase signaling pathways *in vitro* and *in vivo* (33). Chen *et al.* showed that the exosomes derived from cardiac progenitor cells can protect the cardiomyocytes from oxidative stress both *in vitro* and, in IR injury, *in vivo* (43). Human MSCs have an oxidative defense mechanism and resistance to acute apoptosis mediated by ROS (44). Therefore, exosomes of MSCs can indicate an ideal treatment tool for liver disease (18). In terms of limitations, the results may not be completely applied to *in vivo* situations. Thus, it is suggested that these findings will be done on living animal models to confirm their effectiveness in clinical practice and management of the disease.

## Conclusion

In our study, exosome therapy effectively reduced the NOX1, NOX2, and NOX4 mRNA expressions, the phosphorylation of Smad3C protein, and ROS production in TGF-β-treated HSCs. This data showed that human exosomes of WJ-MSCs play the main role in protecting the TGF-β induced HSCs by inhibition of the NOXs pathway and may be a new treatment for liver fibrosis. These results showed that the WJ-MSC exosomes can be used clinically as a regenerative drug in the future.

## Authors’ Contributions

RA and MR designed the study. SSB and ES performed all assays. RA contributed to disease diagnosis and selection of patients. TB analyzed the data. SSB wrote the first draft. RA and ES revised the manuscript. MR and RA contributed to interpreting the results. All authors read and approved the final manuscript.

## Conflicts of Interest

The authors have no conflicting financial interests.
